# Spatial Dynamics and Ecosystem Functioning

**DOI:** 10.1371/journal.pbio.1000378

**Published:** 2010-05-25

**Authors:** Oswald J. Schmitz

**Affiliations:** School of Forestry and Environmental Studies, Yale University, New Haven, Connecticut, United States of America

## Abstract

Ecosystem functioning is dependent upon the way species become distributed across space.

Classical theory of species dynamics in ecosystems is built on the concept of homogeneous, reciprocal interaction. The concept is borrowed from that branch of physics and chemistry dealing with reaction kinetics of molecules in well-mixed gases and liquids. It idealizes individual entities—no longer molecules but now individuals of a species—as interacting with each other or with their predators or competitors in such a way that each individual has an equal likelihood of interacting with every other individual in the system. There is no spatial structure in the system; in fact, space is assumed to be immaterial to system dynamics.

But, any keen observer of nature may cry foul. Unlike the simplified theoretical conception, natural ecosystems are characterized by complex and heterogeneous spatial structure. Plants are clustered into patches. Accordingly, herbivores that eat them and the predators that eat the herbivores become similarly arranged in space [1]. This observation was not lost on ecological theorists who in the 1980s and 1990s began to address spatial heterogeneity more explicitly [1],[2]. This new ecological theory, essentially built on additional concepts from physics and chemistry, (e.g., [3]), partitions system dynamics effectively into two phases: a reaction phase in which individuals of species interact locally and a diffusion phase in which individuals disperse after local interactions take place. Dispersal is activated (a positive feedback) in the reaction phase by factors like intense competition or predation risk that causes individuals to move to less competitive or safer locations. Dispersal becomes inhibited (a negative feedback) whenever individuals' efforts to relocate are rebuffed by individuals already occupying the new locations.

This core reaction–diffusion mechanism has been used to develop two distinct classes of theory for species and ecosystem dynamics. The theories differ fundamentally in assumptions about spatial structure and in the way activation and inhibition feedbacks operate on a landscape. One kind of theory (known now as theory of meta-populations and meta-communities) extends classical theory by imposing spatial patch structure as a physical condition of the system (Figure 1) and recasts parameters describing population birth and death processes in terms of spatial movement processes [4]. It then examines the consequences of that structure on system dynamics through analyses of within-patch species interactions and inter-patch species dispersal [4]. The other kind of theory (known now as theory of self-organized systems) starts with a clean slate and examines how spatial structure emerges as a consequence of species interactions and movements [5]. In the meta-system theory, diffusion across a landscape is inhibited by local, within-patch negative feedback (Figure 1). That is, positive and negative feedbacks operate within local patches [4]. In the self-organized systems theory, the positive feedback is local, and negative feedback manifests itself as tension among local clusters of species that prevents further dispersal (Figure 1). Landscape-scale patch structure thus emerges in self-organized systems theory as a consequence of local positive feedback and landscape-scale inhibitive or negative feedback [5].

**Figure 1:**
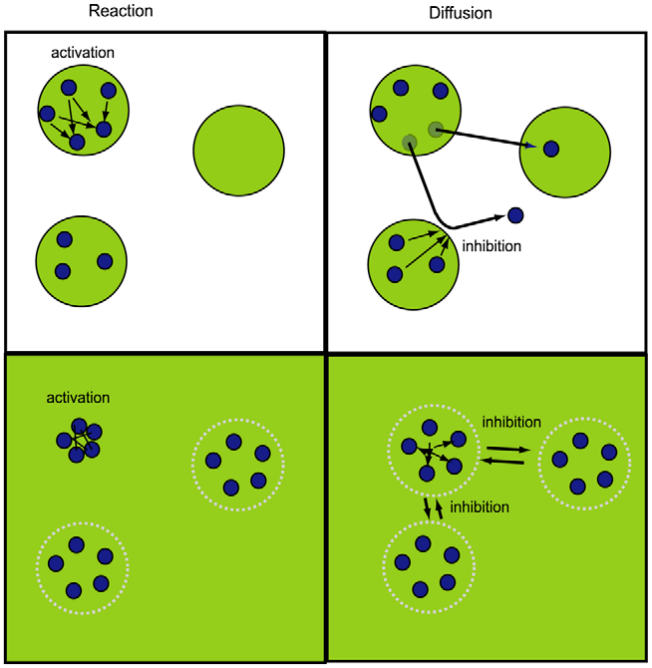
Two alternative representations of spatial dynamics theory for ecosystems. The upper frames represent the mechanisms determining movement of individuals among discrete patches within a landscape. In the simplest idealization of the theory, individuals within discrete patches belong to a local population. Local populations are connected to each other by individual dispersal (diffusion) that is activated by interactions (reaction) among individuals within a patch. Colonization of another patch by individuals is inhibited whenever dense local populations rebuff dispersing individuals. Dispersal connects the dynamics of local populations to create a grand “meta-population.” The bottom frames represent the mechanics of movement of individuals from a local concentration outward (diffusion), again activated by local interactions among individuals. In the simplest idealization of the theory, full, random dispersion of individuals is inhibited whenever individuals encounter and interact with members of other local concentrations within shared buffer zones. This causes regularly spaced clusters of individuals to become self-organized across the landscape.

Meta-systems theory has gained considerable traction in ecology because it resonates with our intuitive understanding of the current state of many ecosystems [6],[7]. For example, small ponds represent natural, discrete patches within terrestrial landscapes, leading to characteristic patterns of local and landscape-scale species abundances and ecosystem functioning [8]. Human activities have also artificially imposed spatial structure onto many ecosystems by fragmenting formerly continuous landscapes into discrete habitat patches. This has led to predictable transformation of species assemblages and their associated functioning owing to differential abilities of species to reside within patches of a particular size and to disperse among them [9]. Meta-systems theory has clear and profound implications for the conservation of biodiversity [6],[7],[10].

The applicability of self-organized systems theory tends to be less clear because it is a more abstract construct than meta-systems theory. Moreover, there is divided opinion as to whether or not the predicted emergent dynamics based on fairly simple mathematical rules of species engagement are robust to changes in assumptions that reflect real-world ecological conditions [11]. This debate, however, continues to be largely academic because the ultimate arbiter—a rich body of empirical evidence from explicit tests of the theory—has not yet been amassed [5]. There certainly are many putative examples of self-organized, large-scale patterns, owing in good part to advances in satellite imagery [5]. And, there have been efforts to resolve mechanisms driving self-organized pattern formation in species populations [12],[13]. But, evidence that such population-level spatial organization influences whole-ecosystem functioning remains a missing piece of the puzzle.

Testing self-organized systems theory in a whole ecosystem context in nature is not an enterprise for those given to do research yielding quick and simple answers. Unlike meta-systems theory there is no easy and fast way to delineate system structure. Patch boundaries of self-organized systems tend to be fuzzy [5], requiring sophisticated statistical techniques to resolve spatial patterning. The success of this kind of analysis is predicated on obtaining an extensive yet finely resolved data set. Before doing that, however, one must decide what a patch is and what drives the patch structure. For example, does patch structure arise from spatial gradients in soil nutrient concentrations that then cause spatial clumping of plants and the build-up of food chains? Or, does the spatial structure emerge from predator–prey interactions that cause species to emanate away from a local point source? More likely, it is a combination of the two, and so, their relative importance must be resolved through strategic experimentation and sampling of biota and physical conditions. Finally, one must find the points of spatial tension and resolve the mechanisms that delineate patch boundaries. The complexity can be perplexing, leading to the ultimate a priori question: where does one begin?

In this issue of *PLoS Biology*, Pringle et al. [14] answer these questions while undertaking a herculean effort to explain the spatial patterning of an African savanna ecosystem. Breakthroughs in our understanding of ecological systems often come from having good understanding of the natural history of the system in question and paying attention to the clues that nature provides [15]. Indeed, Pringle et al. [14] capitalize on important prior natural history clues that there is a tendency for termites to exhibit locally non-overlapping foraging territories around their colonies [16]. Termite movement away from the colony seems to be activated by the need to find food, and movement is eventually inhibited when individual termites encounter and compete with members of another colony [16]. Amazingly, this behavior may lead to quite regular spacing of termite colonies across the landscape [14].

More important to the structure and functioning of the entire savanna ecosystem is that the grass-covered mounds created by termite colonies are sandier than surrounding soils. This allows greater water infiltration, aeration, and nutrient build-up on the mounds relative to surrounding soils [14]. Termite mounds are effectively moisture and nutrient “oases” within a dryland matrix. Concentrated moisture and nutrients accordingly promotes tree species growth at the colony margins, with the thickest trees at the immediate mound perimeter and gradually thinner trees emanating away from the perimeter and intergrading with thin trees emanating from other mounds [14]. The nutrient supplied by the mounds to the trees also fosters the build-up of food chains comprised of insect herbivores and spider and lizard predators of the insects.

This emergent structure also leads to parallel activation and inhibition dynamics among species in the food chain [14]. The herbivorous insects are highly concentrated on thicker trees near the mounds and decrease in abundances on thinner, distant trees. Lizards and spiders are likewise dispersed, and field experimentation showed that this was partly because thicker trees offered better hunting sites and partly because prey density was highest on thick trees that tended to be closets to the mounds. A related study [17] shows that nitrogen content of plants is higher near termite mounds, too, meaning that both food quantity and quality is higher near mounds, which likely contributes to all of these patterns.

The combination of nutrient supply for primary plant production, and the translation of plant nutrients into herbivore and predator secondary production mean that termite mounds also become hotspots of ecosystem productivity. These hotspots are preserved through the interplay between activation and inhibition of spatial movement of all of the components of the ecosystem. Thus, the landscape displays a regular pattern of high and low productivity that mirrors the regular patterning of termite mounds. Further statistical modeling suggests that this form of heterogeneity results in greater net productivity than would be expected if the termite mounds were irregularly clustered across the landscape [14]. This derives from the statistical property that when patches are regularly spaced, no single point is very far from a mound, so the productivity of all points when averaged is greater than would be the case when patches are highly clustered or randomly dispersed [14]. Of course, it would be exceedingly difficult to execute the definitive experimental test of this assertion, which would require rearranging the spatial configuration of the termite mounds. This is perhaps the biggest Achilles heel of any empirical effort to test self-organized systems theory within a real-world ecosystem. Nonetheless, the study [14] is exemplary in that it comes the closest yet to satisfying empirical conditions needed to demonstrate the existence of a self-organized ecosystem [5]. By amassing animal behavioral, animal population, and ecosystem data, the authors thus provide a reasonably coherent picture of the spatial mechanisms driving ecosystem structure and functioning.

The intriguing thing is that if, instead of focusing down on a neighborhood of termite mounds, we took a bird's-eye aerial view of the landscape, we could be fooled into concluding that a savannah is a fairly homogeneous landscape. And indeed it is quite plausible to draw such a view of system structure given increased and widespread application of modern satellite imagery to study patterning of savannas and other ecosystems [5],[18],[19]. Then again, if we focused too closely on a termite mound and just its immediate surroundings, our perspective might become so overwhelmed by highly resolved local species interactions that we risk not seeing the spatial patterning at all. The art in empirically resolving the structure and dynamics of self-organized ecosystems is deciding on the appropriate scale of resolution for study [1],[5]. This is not a trivial exercise because it requires years of intrepid field research aimed at understanding both the natural history of a system, and measuring spatial pattern and dynamical processes at many different but complementary spatial perspectives. This may well be the single most important reason why more field evidence for self-organized systems is not yet available.

The study by Pringle et al. [14] nicely shows that theory of self-organized systems is not merely a virtual computer-world phenomenon. There is indeed a basis in “robust reality” [11]. Like meta-systems theory, the implications of self-organized systems theory for conservation, as demonstrated by the study [14], are profound. In this particular case, a very non-charismatic species of fungus-cultivating termite that lives predominantly below-ground seems to create biophysical and biotic conditions that lead to the evolution of aboveground trophic structure and parallel self-organized dynamics in the higher trophic levels. This would, in turn, suggest that the loss of any one of the parts would cause the parallel dynamics sustaining overall ecosystem functioning to quickly collapse. This reinforces the need to consider how the nature of species interactions link to whole-ecosystem functioning when developing strategies to conserve biodiversity [7],[15],[20].
